# Balloon-oriented puncture for creating an access for endovascular aortic aneurysm repair in a case of iliac and femoral artery occlusion

**DOI:** 10.1186/s42155-020-00116-3

**Published:** 2020-05-11

**Authors:** Shigeo Ichihashi, Satoru Nagatomi, Shinichi Iwakoshi, Masahiro Inagaki, Francesco Bolstad, Kimihiko Kichikawa

**Affiliations:** 1 Department of Radiology, Nara Medical University, 840 Shijyocho, Kashihara, Nara 634-8521 Japan; 2 Department of Clinical English, Nara Medical University, 840 Shijyocho, Kashihara, Nara 634-8521 Japan

**Keywords:** Aortic aneurysm, Abdominal, Endovascular procedures, Peripheral arterial disease

## Abstract

**Background:**

Abdominal aortic aneurysms (AAA) with iliac artery occlusive diseases are not uncommon. When an occlusion extends from iliac artery to common femoral artery (CFA), adjunctive procedures such as endareterectomy of CFA and angioplasty of iliac artery are performed prior to endovascular aneurysm repair (EVAR). Alternatively, aorto-uni-iliac stentgrafting with femoro-femoro bypass surgery could be performed. If run off vessels such as superficial femoral artery (SFA) and profunda femoris artery (PFA) are both occluded in addition to the CFA, surgical procedures may become extremely complex, with much longer procedure time. We present an unusual case of AAA with arterial occlusion ranging from external iliac artery (EIA) to superficial and profunda femoris arteries, which was fully managed with endovascular means.

**Case presentation:**

The patient was a 76 year old male who was found incidentally to have a fusiform infrarenal AAA, the size of which was 55 mm in maximal transverse diameter. Despite the occlusions of left EIA, CFA and proximal parts of SFA and PFA, he did not have ischemic symptoms in his left leg due to the development of abundant collateral networks from left internal iliac artery. The patient had a past history of endarterectomy of left CFA. Since a repeated endarterectomy or interposition grafting of the CFA were deemed extremely difficult, without any patent runoff vessel, EVAR was performed using the occluded vessel simply as a conduit for the delivery of the endograft, without revascularizing the vessel. An angioplasty balloon was delivered from right CFA to the occluded left CFA through a subintimal space. A percutaneous puncture of the expanded balloon was done at the occluded left CFA under fluoroscopy, inserting the guidewire into the punctured balloon, finally establishing the through and through wire. EVAR was successfully performed using AFX unibody stentgraft without any complication.

**Conclusion:**

AAA with access vessel occlusions from EIA to SFA was successfully treated with EVAR with the aid of the balloon oriented percutaneous puncture technique. Having the technique as an armamentarium can broaden the application of EVAR for AAA with the complicated access.

## Background

Endovascular aneurysm repair (EVAR) is a less invasive therapeutic option for abdominal aortic aneurysm (AAA) compared to open surgery and is being increasingly performed worldwide (Chadi et al. 2012). The feasibility and outcomes of EVAR are highly dependent on various anatomic factors, including diameter or patency of the iliac access route. In previous studies, AAA with coexisting iliac occlusive disease reportedly precluded EVAR in 6–15.4% of patients (Arko et al. 2004). Nevertheless, an advancement of medical devices and increased operators’ experiences have lead to high technical success rates even in AAA with occlusive iliac access (Uchiyamada et al. 2013; Vallabhaneni et al. 2012; Takeuchi et al. 2019). In cases of iliac artery occlusion extending to the common femoral artery (CFA), an ancillary endarterectomy/ interposition grafting of CFA is generally required. However, such an ancillary surgery is difficult when runoff vessels, i.e. superficial femoral artery (SFA) or profunda femoris artery (PFA), are occluded. Herein a case of AAA with a coexisting access route occlusion from the external iliac artery (EIA) to the SFA is presented where a technique of percutaneous puncture of angioplasty balloon was utilized to establish the through and through wire prior to EVAR.

## Case presentation

**Fig. 1:**
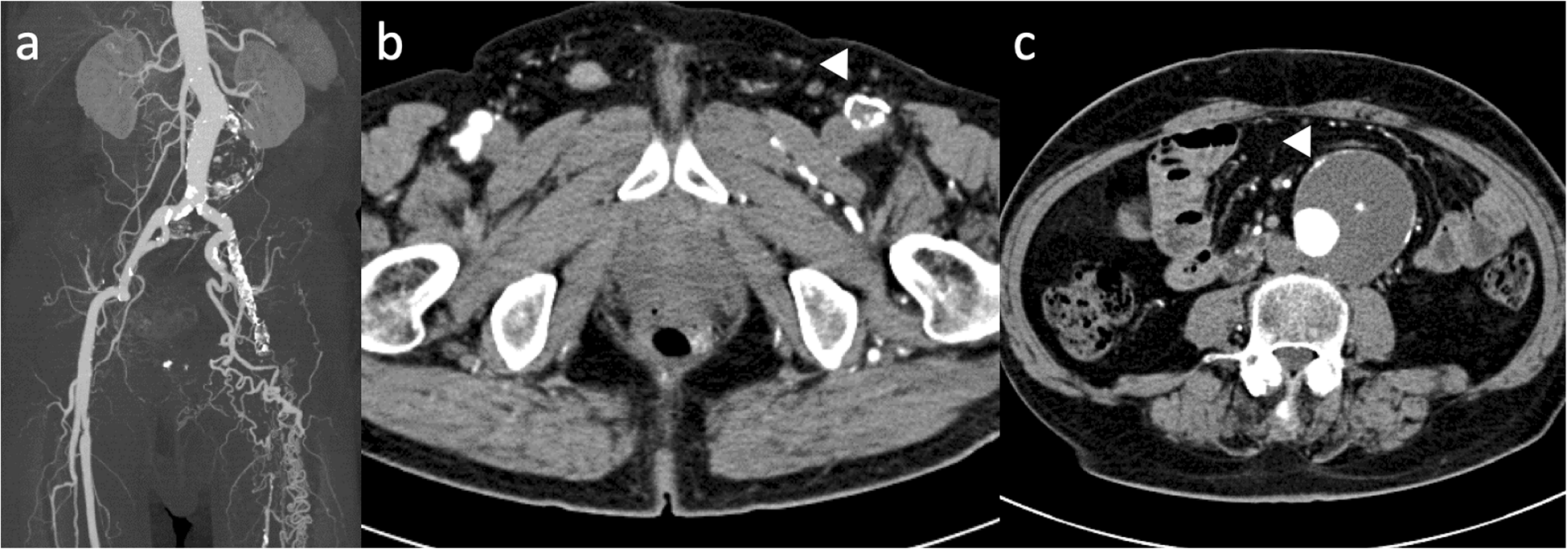
CT before endovascular aneurysm repair. **a**: MIP image of CT angiography shows the occlusion of left EIA to SFA with abundant collateral channels from left hypogastric artery, **b**: transaxial image shows the occluded CFA with circumferential vessel wall calcification (arrow head), **c**: abdominal aortic aneurysm with the diameter of 55 mm (arrow head). CFA: common femoral artery, EIA: external iliac artery, MIP: maximum intensity projection, SFA: superficial femoral artery

**Fig. 2:**
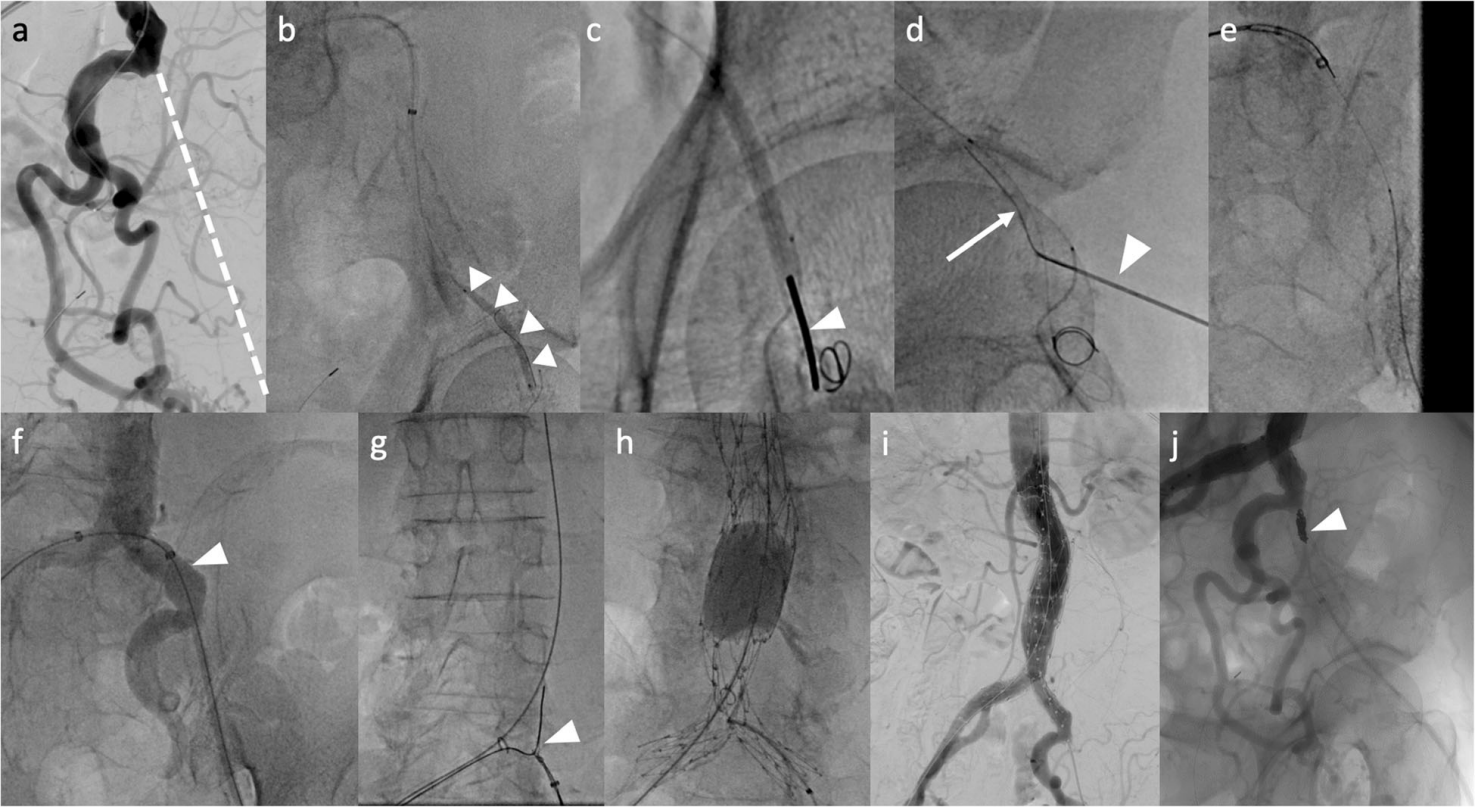
Procedure detail. **a**: The angiogram shows the occlusion of left EIA (dotted line), **b**: An angioplasty balloon was advanced to the occluded CFA and expanded (arrow heads), **c**: Percutaneous puncture of expanded balloon at the occluded CFA (arrow head), **d**: A guidewire (arrow) was inserted into the punctured balloon (arrow head), **e**: A through and through wire was established, **f**: A 8F sheath was advanced to the left CIA over the through and through wire (arrow head), **g**: the contralateral wire attached to the endograft was caught by a snare catheter (arrow head) advanced from the left CFA, **h**: After endograft deployment, a touch-up balloon was expanded inside the endograft, **i**: Completion angiogram shows a successful exclusion of AAA, **j**: Coil embolization was performed to occlude the tract where the 8F sheath was advanced (arrow head). AAA: abdominal aortic aneurysm, CFA: common femoral artery, CIA: common iliac artery, EIA external iliac artery

**Fig. 3:**
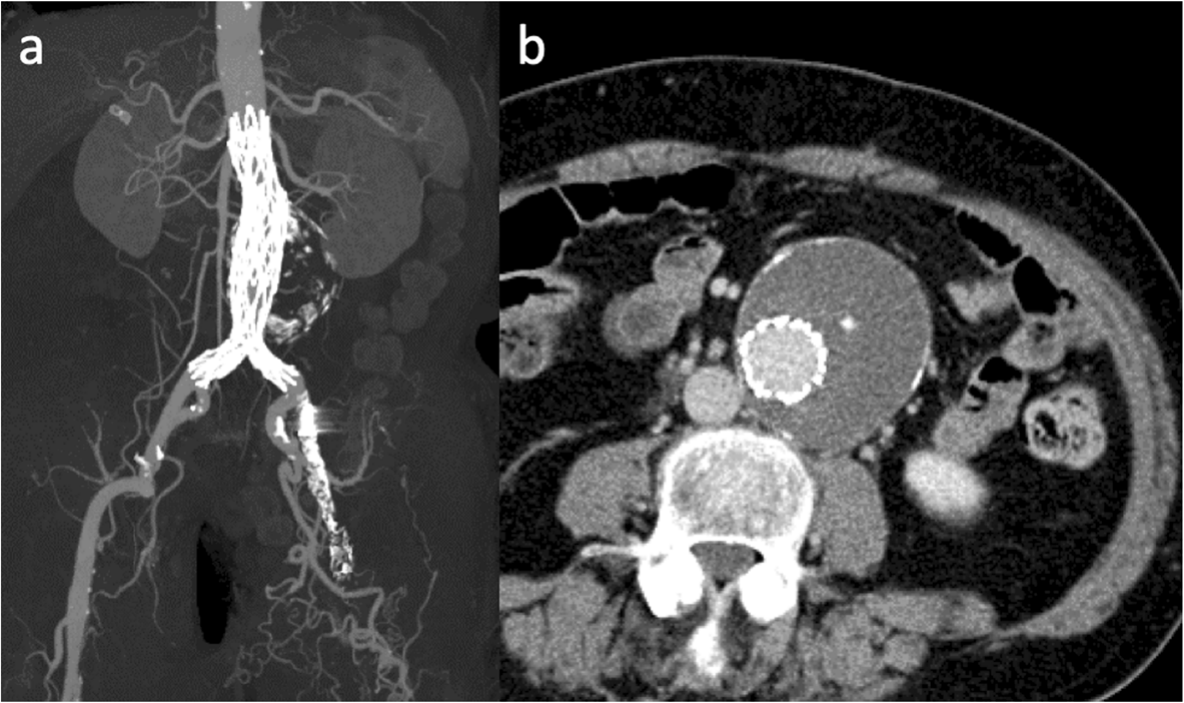
Post operative CT. **a**: MIP image of CT angiography shows an implanted stentgraft in the aorta with the bilateral legs deployed in the CIA, **b**: AAA was successfully sealed without any visible endoleak. AAA: abdominal aortic aneurysm, CIA: common iliac artery, MIP: maximum intensity projection

The case was a 76-year-old man affected by a 55 mm infrarenal AAA (Fig. 1). All of the left EIA, CFA and SFA were totally occluded. He had a past history of undergoing endarterectomy of the left CFA. The collateral circulation was well developed from the left internal iliac artery (IIA) through the PFA, and the patient did not complain of any ischemic symptom in his leg, corresponding to the Rutherford grade 0. Since a repeated endarterectomy or interposition grafting of the CFA was deemed extremely difficult, without any patent runoff vessel, it was decided to perform an EVAR using the occluded vessel simply as an endovascular conduit for the delivery of the endograft, without revascularizing the vessel (Fig. 2). First, the occluded left CFA was punctured under ultrasound guidance. Initially, an attempt was made to advance a guidewire (0.014 in. Astato XS 9–40, Asahi Intec, Nagoya, Japan) retrogradely, however the guidewire went outside of the occluded EIA. After another access was obtained from the contralateral (right) CFA, another guidewire (0.014 in. Gradius, Asahi Intecc, Nagoya, Japan) was finally able to be advanced down to the occluded left CFA antegradely via subintimal lumen of the occluded left EIA. An angioplasty balloon (JADE 2.5 mm/40 mm, OrbusNeich, Hong Kong) was subsequently delivered to the occluded left CFA and dilated. A percutaneous puncture of the expanded balloon with a 21 gage needle (Micropuncture access set: COOK Medical, Bloomington, IL, USA) was performed at the occluded left CFA under fluoroscopy, followed by the insertion of a guidewire (0.014 in. Cruise, Asahi Intecc, Aichi, JAPAN) into the punctured balloon. The punctured balloon was slowly retracted into the sheath advanced from the contralateral CFA, finally establishing the through and through wire. A 8F sheath was advanced from the occluded left CFA to the left common iliac artery (CIA) over the through and through wire. While the main body of the AFX 2 endograft (Endologix Inc., Irvine, CA, USA) was being delivered to the aorta via the right iliac artery, the contralateral wire attached to the endograft was caught by a snare catheter (Indy OTW Vascular Retriever, COOK medical, Bloomington, IN, USA) and withdrawn out of the 8F sheath advanced from the occluded left CFA. After deployment of the endograft in the aorta, the contralateral limb of the endograft was subsequently deployed in the left CIA. A completion angiogram demonstrated successful exclusion of AAA without any visible endoleak. Since there was a residual antegrade flow into the left EIA where the 8F sheath had been advanced, embolization using coils was performed in order to close the channel used as the endovascular conduit. Contrast enhanced CT 1 week after EVAR showed the successfully sealed AAA without sacrificing collateral channels from left IIA (Fig. 3).

## Discussion

The EVAR was successfully performed for AAA with unilateral iliac access occlusion extending to the SFA using the percutaneous balloon puncture technique. With this technique, as long as the balloon can be advanced down to the CFA, the through and through wire can be reliably established by simply puncturing the balloon under fluoroscopic guidance. This technique was reported for the insertion of tunneled hemodialysis catheters (Too et al. 2016). Too et al. reported ten cases of the catheter insertion via occluded veins using the technique, with a technical success rate of 100%. A snare catheter can be utilized instead of balloons to be punctured, followed by grabbing the guidewire inserted via needle. Testi et al. reported that a balloon which was advanced subintimally from distal anterior tibial artery was puncture at CFA to revascularize the occluded SFA (Testi et al. 2019). The current case was the first one using the technique prior to EVAR. The biggest advantage of the technique is that it can be performed even when the puncture site is occluded.

Technical successes of the EVAR for AAA with coexisting iliac artery occlusion are reportedly high with a success rate of 87–100% (Uchiyamada et al. 2013; Vallabhaneni et al. 2012; Takeuchi et al. 2019). For occlusions extending from the iliac artery to the CFA, endovascular revascularization plus concomitant endarterectomy of the CFA can be performed (Takeuchi et al. 2019). However, in cases like the one reported here, revascularization can be difficult due to the past history of endarterectomy and the lack of distal patent vessels. Additionally, ischemic symptom of the limb was not observed owing to the abundant collateral channels via IIA and PFA. On the other hand, use of an aorto-uniiliac graft, which could be an alternative option for the occluded iliac access, might sacrifice the arterial flow into left IIA and could lead to sever limb ischemia in this case.

There could be a debate as to whether the embolization of the tract at the end of the procedure was necessary. Although manual compression of the CFA with/without the usage of a vascular closure device might have been sufficient for closing the channel, further experience is needed on which method is more suitable to prevent the hemorrhagic complication from the groin.

## Conclusions

AAA with access vessel occlusions from EIA-SFA was successfully treated with the balloon oriented percutaneous puncture technique at the occluded CFA. The puncture of the expanded balloon is very simple and can be performed at any occluded vessel. Having the technique as an armamentarium can broaden the application of EVAR for AAA with the complicated access such as occluded CFA.

## Data Availability

Not applicable.

## Abbreviations

AAA: Abdominal aortic aneurysm
CFA: Common femoral artery
CIA: Common iliac artery
EIA: External iliac artery
EVAR: Endovascular aneurysm repair
IIA: Internal iliac artery
PFA: Profunda femoris artery
SFA: Superficial femoral artery
